# Reconstruction of Bacterial Metagenome-Assembled Genome Sequences from Alpine Bog Vegetation

**DOI:** 10.1128/MRA.00821-20

**Published:** 2020-08-27

**Authors:** Wisnu Adi Wicaksono, Tomislav Cernava, Christian Berg, Gabriele Berg

**Affiliations:** a Institute of Environmental Biotechnology, Graz University of Technology, Graz, Austria; b Institute of Plant Sciences, University of Graz, Graz, Austria; Loyola University Chicago

## Abstract

Bacteria are essential constituents of bog ecosystems. Here, we report 44 bacterial genome sequences reconstructed from metagenomes sampled across 12 plant species representing Alpine bog vegetation. This resource will facilitate further exploration of the genetic potential of these bacteria and allow researchers to refine their ecological roles in association with their plant hosts.

## ANNOUNCEMENT

The bog ecosystem is known as the oldest vegetation form and plays an important role in the global carbon cycle and storage (1–3). It is commonly dominated by *Sphagnum* mosses that have an ombrotrophic lifestyle and depend on their associated microbiome (4, 5). Previous studies have shown that *Sphagnum* mosses harbor a specific bacterial community which fulfils important functions, i.e., nutrient supply and pathogen defense (5–9). Keystone microbes within bog vegetation were shown to be present in typical naturally occurring plants and formed a mutual metacommunity embedded in the predominant *Sphagnum* species (10). Despite numerous studies on this globally important ecosystem, we lack detailed insights into the genomes of the highly diverse bog-associated bacteria (7, 10) that are at present largely underrepresented in global surveys. Here, we report the binned metagenomic coassembly of 12 metagenome samples obtained from different plant species in a representative Alpine bog vegetation ecosystem.

Twelve plant species representing the natural vegetation of Alpine bog ecosystems were previously obtained from two *Sphagnum*-dominated plots (1 m^2^ each) in Rotmoos and Pürgschachen Moor in Austria (10–12). Total genomic DNA was extracted from 5 g of each plant species using the FastDNA spin kit for soil (MP Biomedical, USA). The total community DNA was sent to the sequencing provider Eurofins MWG Operon (Ebersberg, Germany), and sequencing libraries were prepared using a TruSeq DNA library kit. Paired-end sequence reads (2 × 150 bp) were generated using a HiSeq 2500 system (Illumina, US) resulting in a range of 22.7 to 40.9 million reads per individual metagenome.

Default parameters were used for all software unless otherwise noted. Trimmomatic v0.39 and VSEARCH v2.14.2 were used to remove Illumina adapter and low-quality reads (Phred, <20), respectively. Metagenomic data sets were coassembled using MEGAHIT v1.2.9 with metasensitive parameters (13), resulting in 22,967 metagenome contigs with a length of >1 kbp. The metagenomic contigs were binned using Maxbin2 v2.2.7, MetaBAT2 v2.12.1, and CONCOCT v1.1.0 (14–16) and dereplicated into metagenome-assembled genomes (MAGs) using DAS Tool v1.1.1 (17). CheckM v1.0.13 was used to calculate genome coverage, completeness, and the percentage of contaminations in the MAGs (18). The quality of MAGs was defined according to the current definition of the minimum information metagenome-assembled genome (MIMAG) standards (19). Taxonomical classification of each MAG was obtained using the Bin Annotation Tool (BAT) v4.6 (20).

A total of 44 MAGs with a contamination level lower than 10% were recovered and assigned to the following bacterial phyla: *Proteobacteria* (17 MAGs), *Acidobacteria* (8 MAGs), *Actinobacteria* (7 MAGs), *Verrumicrobia* (4 MAGs), and *Chlamydiae* (2 MAGs) (Table 1). Moreover, 6 MAGs were classified to the candidate phylum *Saccharibacteria* (2 MAGs), *Eremiobacteraeota* (2 MAGs), and *Melainabacteria* (2 MAGs). Among the whole collection, 18 MAGS were classified as high quality, 22 as medium quality, and 4 as low quality. The estimated completeness rates of the recovered MAGs were in a range of 4.8% to 97.6%, the genome sizes in a range of 493,724 to 6,564,149 bp, and the GC contents in a range of 36.6% to 73.2%. These metagenome-assembled genomes will provide deeper insights into the genetic reservoir of plant-associated microbial communities of Alpine bogs that play important roles for ecosystem services and must be considered in biodiversity conservation.

**TABLE 1:** Detailed taxonomic classification, completeness and contamination values, genome sizes, GC content, MIMAG status, taxonomy, and ENA accession numbers of bacterial MAGs

MAG alias	Taxonomic classification	No. of contigs	Genome coverage (×)	Completeness (%)	Contamination (%)	Genome size (bp)	GC content (%)	MIMAG classification	ENA accession no.
BOG_genome_mining.154	*Rhodospirillales*	51	192	97.6	2.1	4,858,929	60.8	High	CAJCIG010000000
BOG_genome_mining.57	*Verrucomicrobia*	133	19	97.2	5.6	3,678,110	57.9	High	CAJCIP010000000
BOG_genome_mining.2	*Gammaproteobacteria*	201	49	97.0	0.6	5,517,870	61.4	High	CAJCIO010000000
BOG_genome_mining.201	*Acidobacteriaceae*	319	32	96.8	3.0	5,452,513	59.6	High	CAJCIT010000000
BOG_genome_mining.200	*Beijerinckiaceae*	263	10	96.7	1.9	5,352,509	47.8	High	CAJCIQ010000000
BOG_genome_mining.129	*Gammaproteobacteria*	133	8	96.6	8.0	3,708,935	61.3	High	CAJCIC010000000
BOG_genome_mining.136	*Rhodanobacteraceae*	151	30	96.4	7.0	4,133,772	63.1	High	CAJCHN010000000
BOG_genome_mining.119	*Mycobacteriaceae*	135	6	96.2	3.3	3,466,724	61.9	High	CAJCHO010000000
BOG_genome_mining.39	*Acidobacteria*	293	23	96.1	3.7	4,289,667	64.1	High	CAJCIS010000000
BOG_genome_mining.9	“*Candidatus* Saccharibacteria”	56	18	95.7	1.3	1,972,358	60.5	High	CAJCJA010000000
BOG_172_sub	*Betaproteobacteria*	607	7	95.1	9.2	6,564,149	64.9	High	CAJCHU010000000
BOG_genome_mining.134	*Betaproteobacteria*	131	19	93.6	4.7	5,607,130	57	High	CAJCHZ010000000
BOG_genome_mining.99	*Myxococcales*	279	1	93.0	5.7	2,944,536	39.8	High	CAJCIZ010000000
BOG_genome_mining.110	*Rhodospirillales*	329	8	92.8	1.7	4,334,065	62.2	High	CAJCID010000000
BOG_genome_mining.93	“*Candidatus* Eremiobacteraeota”	247	10	92.6	6.2	4,528,035	59.6	High	CAJCJD010000000
BOG_genome_mining.12	*Acidobacteria*	91	28	92.4	1.4	2,092,162	43.1	High	CAJCHM010000000
BOG_genome_mining.22	*Rhodospirillales*	312	17	91.5	2.9	3,260,124	67.2	High	CAJCIN010000000
BOG_194	*Acidobacteriaceae*	894	1	91.4	6.4	4,992,893	68.3	High	CAJCHX010000000
BOG_genome_mining.142	*Chlamydiae*	214	15	89.0	3.7	2,655,961	66.9	Medium	CAJCHW010000000
BOG_genome_mining.111	*Actinobacteria*	525	14	88.4	6.1	4,090,219	65.4	Medium	CAJCHR010000000
BOG_genome_mining.63	*“Candidatus* Saccharibacteria”	393	6	88.3	2.1	3,137,154	36.6	Medium	CAJCJB010000000
BOG_genome_mining.10	*Betaproteobacteria*	271	17	88.1	6.4	2,926,007	63.8	Medium	CAJCIA010000000
BOG_genome_mining.178	*Bacteroidetes*	243	17	88.0	3.0	2,882,424	59.7	Medium	CAJCII010000000
BOG_genome_mining.24	“*Candidatus* Melainabacteria”	350	16	87.1	1.9	2,597,317	73.2	Medium	CAJCIY010000000
BOG_maxbin_BOG.405	*Actinobacteria*	341	2	86.7	7.5	1,828,578	42	Medium	CAJCJF010000000
BOG_genome_mining.144	“*Candidatus* Eremiobacteraeota”	270	10	83.3	7.0	4,296,633	63.2	Medium	CAJCIB010000000
BOG_genome_mining.202	*Acidobacteriaceae*	214	45	83.2	0.9	3,824,088	60.2	Medium	CAJCIM010000000
BOG_genome_mining.146	*Actinomycetales*	595	12	80.6	6.7	3,343,448	71.8	Medium	CAJCHV010000000
BOG_286	*Bryobacterales*	1,072	7	78.5	2.6	6,535,982	50.7	Medium	CAJCHQ010000000
BOG_genome_mining.30	*Verrucomicrobia*	257	7	75.3	2.7	1,650,402	46	Medium	CAJCIW010000000
BOG_genome_mining.92_sub	“*Candidatus* Melainabacteria”	1,020	9	73.5	3.4	4,860,993	68.8	Medium	CAJCJE010000000
BOG_genome_mining.192_sub	*Actinobacteria*	379	8	71.2	2.4	2,058,861	68.1	Medium	CAJCIJ010000000
BOG_genome_mining.15	Nevskia soli	455	14	71.1	3.1	4,156,146	59.3	Medium	CAJCHS010000000
BOG_136	*Rhizobiaceae*	805	5	70.4	5.7	1,818,679	46.1	Medium	CAJCHY010000000
BOG_genome_mining.3	*Chlamydiae*	74	7	70.3	0.0	802,801	43.5	Medium	CAJCIR010000000
BOG_genome_mining.73	*Alphaproteobacteria*	502	9	67.3	1.3	2,586,371	62.3	Medium	CAJCJC010000000
BOG_267_sub	*Acidobacteriaceae*	476	2	65.9	4.7	1,629,168	45.1	Medium	CAJCHP010000000
BOG_genome_mining.164_sub	*Actinobacteria*	548	1	65.3	0.9	3,748,987	47	Medium	CAJCIF010000000
BOG_genome_mining.207	*Acidobacteriaceae*	411	9	62.9	3.3	3,201,545	65.1	Medium	CAJCIV010000000
BOG_genome_mining.187	*Verrucomicrobia*	1,023	6	54.6	2.2	4,462,797	69.9	Medium	CAJCIK010000000
BOG_genome_mining.171	*Sphingomonadaceae*	207	4	45.3	1.1	858,991	62.8	Low	CAJCIH010000000
BOG_200	*Gammaproteobacteria*	616	3	34.6	4.1	846,789	56.1	Low	CAJCIE010000000
BOG_genome_mining.18	*Verrucomicrobia*	370	7	30.3	1.0	1,392,061	68.9	Low	CAJCIL010000000
BOG_genome_mining.31	*Pseudonocardiaceae*	69	1	4.8	0.0	493,724	53.9	Low	CAJCIU010000000

### Data availability.

This shotgun metagenome project has been deposited in the European Nucleotide Archive (ENA) database under the study number PRJEB39100 and accession numbers ERR4298333 and ERR4298344. The MAG sequences are accessible in the ENA repository under the accession numbers provided in Table 1.
